# Effects of Dietary Concentrate-to-Roughage Ratio on Rumen Microbiota, Functional Profiles, and Fermentation Characteristics in Yak

**DOI:** 10.3390/microorganisms14061223

**Published:** 2026-05-29

**Authors:** Fajie Gou, Qingye Zhao, Yincang Han, Yonggang Sun, Weiqin Ding, Jianyu Chen, Shengwei Jin

**Affiliations:** 1 Academy of Animal Science and Veterinary Medicine, Qinghai University, Xining 810016, China; 2 Key Laboratory of Plateau Livestock Genetic Resources Protection and Innovative Utilization of Qinghai Provincial, Xining 810016, China; 3 Qinghai Livestock and Poultry Genetic Resources Protection and Utilization Center, Xining 810000, China

**Keywords:** concentrate-to-roughage ratio, yak, rumen microbiota, metagenomics, rumen fermentation factors

## Abstract

This study investigated the effects of different concentrate-to-roughage ratios on the rumen microbial community, functional potential, and fermentation characteristics in yak. Forty Qinghai Plateau-type yaks (8–9 months, 68.725 ± 18.973 kg) were randomly assigned to four dietary groups with concentrate-to-roughage ratios of 80:20 (C80), 65:35 (C65), 50:50 (C50), and 35:65 (C35). After a 15-day adaptation period, animals were fed for 105 days. Rumen contents were analyzed using metagenomic sequencing combined with fermentation parameter measurements. High-concentrate diets (C80 and C65) were associated with increased relative abundance of starch-degrading and propionate-producing bacteria, such as *Prevotella* and *Succiniclasticum*, whereas low-concentrate diets (C50 and C35) were associated with higher abundance of cellulolytic bacteria, including *Ruminococcus* and *Fibrobacter*. Functional analysis indicated increased relative abundance of genes involved in glycolysis (ko00010), propanoate metabolism (ko00640), and energy-related pathways in high-concentrate groups, while fiber degradation and methane-related pathways were relatively higher in low-concentrate groups. Rumen fermentation parameters showed a significant decrease in pH with increasing concentrate level (*p* = 0.001), and NH_3_-N concentrations differed among treatments (*p* = 0.036). Dietary concentrate-to-roughage ratio significantly influences rumen microbial composition, functional potential, and fermentation characteristics in yak. A moderate concentrate level (approximately 65:35) may contribute to a more balanced rumen microbial and fermentation profile under the conditions of this study.

## 1. Introduction

The nutritional efficiency of ruminants directly influences both the economic benefits of animal husbandry and the sustainability of livestock production systems. Ruminants play a key role in converting non-edible biomass into high-quality nutrients, contributing to food security and rural livelihoods [1]. In ruminant production, the dietary concentrate-to-roughage ratio is a key determinant of feed efficiency, rumen microbial community characteristics, and overall animal performance [2]. With the intensification of farming systems in recent years, concentrate proportions in feed formulations have generally increased to support high production performance. However, excessive concentrate intake has raised widespread concern due to its association with ruminal acidosis and impaired ruminal function [3,4]. The substitution of cereal-based concentrates with fibrous by-products has been proposed as a strategy to improve sustainability while maintaining rumen function [5]. Thus, understanding the mechanisms through which dietary concentrate-to-roughage ratios regulate the rumen environment, microbial community structure, and function has become an urgent and essential topic in the fields of animal nutrition and ruminant ecology.

The stability and diversity of rumen microbial communities play a fundamental role in maintaining host health and production efficiency. Previous studies have demonstrated that rumen microorganisms are critical for feed conversion, fiber degradation, protein and nitrogen metabolism, and energy supply [6,7]. High-concentrate diets induce marked shifts in microbial phylum abundances and are often associated with enrichment in pathways such as glycosaminoglycan degradation, apoptosis, and ECM–receptor interactions compared with low-concentrate diets, indicating that the dietary concentrate-to-roughage ratio substantially modulates rumen microbial composition and functional activity [8]. High-concentrate diets typically reduce rumen pH, promote lactic acid accumulation, and decrease fiber-degrading bacterial abundance, leading to microbial dysbiosis. In contrast, higher roughage ratios favor fiber-degrading microorganisms and enhance ruminal fiber degradation capacity [9,10]. Excessive intake of rapidly fermentable carbohydrates has been shown to induce digestive imbalance and altered growth patterns in young ruminants under field conditions [11]. Nevertheless, the mechanisms by which concentrate-to-roughage ratios regulate rumen microbial community structure, metabolic potential, and their relationships with fermentation parameters remain insufficiently understood, particularly at the species and functional pathway levels.

Advancements in metagenomic technology provide a powerful approach for elucidating the mechanisms underlying shifts in rumen microbial composition and metabolic function. This technology enables comprehensive characterization of microbial species composition while allowing in-depth investigation of functional gene profiles and metabolic pathways. Using multi-omics, Ma et al. [12] reported that different dietary energy levels significantly influenced yak rumen microbial abundance and cellulose degradation-related functions (e.g., GH, CE). Similarly, Wang et al. [13] demonstrated through metagenomic analysis that diets with varying roughage-to-concentrate ratios significantly affect the abundance of cellulose-degrading enzymes (CAZymes) in dairy cows, thereby regulating rumen fermentation parameters and feed utilization efficiency. However, research integrating rumen microbial structure, functional pathways, and fermentation parameters under different roughage-to-concentrate ratios remains limited. Therefore, the present study employs metagenomic sequencing combined with multifunctional databases—including NR, KEGG, CAZy, and COG—to comprehensively investigate the effects of different concentrate-to-roughage ratios on rumen microbial community structure and functional composition. By identifying differential species and metabolic pathways and conducting environmental correlation analyses with rumen fermentation parameters, this study aims to reveal the association patterns and mechanistic links between microbial community shifts and rumen environmental changes. These findings will clarify the ecological mechanisms by which rumen microorganisms respond to variations in dietary concentrate-to-roughage ratios and provide a theoretical foundation for optimizing nutritional strategies in ruminant production.

## 2. Materials and Methods

### 2.1. Experimental Animals and Design

At the conclusion of the feeding trial, all yaks were transported to a licensed commercial slaughter facility located approximately 75 km from the experimental site. Transportation was conducted early in the morning to minimize stress, and animals were allowed to rest for 12 h in holding pens with free access to water but no feed. To ensure animal welfare and compliance with ethical standards, all yaks were slaughtered under the supervision of professional slaughterhouse staff in accordance with the guidelines set by the China National Standards for the Humane Slaughter of Livestock [14] and approved by the Animal Ethics Committee of the Institutional Animal Care and Use Committee of Qinghai University, with approval number 2023-QHMKY-001. Each yak was individually restrained and rendered unconscious using a captive bolt stunning device (non-penetrating type), which was applied to the frontal region of the skull. Immediate loss of consciousness was confirmed by the absence of corneal reflex, rhythmic breathing, and response to pain stimulus. Once unconscious, the animal was suspended and exsanguinated by severing the carotid arteries and jugular veins using a sharp knife. This method ensured rapid and complete bleeding, and no animals regained consciousness during the procedure. The entire process was monitored by trained personnel to ensure strict adherence to animal welfare protocols and to minimize suffering.

This study was conducted from January to May 2024 at the Bianma Meron Palm Cooperative in Qilian County, Haibei Tibetan Autonomous Prefecture, Qinghai Province, China (38°45′61″ N, 99°54′27″ E and with average altitude 3450 m). Forty Qinghai Plateau-type yaks aged 8–9 months, with good body condition and similar body weights (68.725 ± 18.973 kg), were selected and randomly assigned to four groups, each with 10 replicates. The treatment groups were fed total mixed rations (TMRs) with concentrate-to-roughage ratios of 80:20 (C80), 65:35 (C65), 50:50 (C50), and 35:65 (C35), respectively. The experimental period consisted of a 15-day pre-trial followed by a 105-day main trial. Diets were formulated according to the Chinese Beef Cattle Feeding Standards [15], and diet composition and nutrient levels are presented in Table 1. Prior to the trial, housing facilities were cleaned and disinfected. All yaks were ear-tagged according to group assignment before entering the pens. Animals were fed twice daily at 09:00 and 17:00, ensuring that feed remained after each meal. Daily leftover feed was weighed before the following morning’s feeding. Throughout the experiment, all yaks had ad libitum access to feed and water. 

### 2.2. Rumen Sample Collection and Analysis

Six yaks were randomly selected from each group and fasted without water for 24 h prior to slaughter. The rumen, reticulum, omasum, and abomasum were separated using sutures to prevent reflux of chyme between adjacent compartments. Rumen fluid was collected by discarding an initial portion, followed by the extraction of 80 mL of rumen fluid. The sample was filtered through four layers of non-woven gauze, and pH was measured immediately using a portable pH meter (PHS-3C, Leijun Technology Co., Ltd., Beijing, China). Ammonia nitrogen (NH_3_-N) content was determined using a blood ammonia assay kit (Nanjing Chengjian Biological Engineering Research Institute, Nanjing, China), following the manufacturer’s instructions. Volatile fatty acids (VFAs) were measured using an Agilent Technologies 6890N-GC gas chromatograph (Agilent, Santa Clara, CA, USA), and bacterial cellular protein (BCP) content was determined using a spectrophotometer. Remaining rumen fluid was aliquoted into 10 mL centrifuge tubes and stored in liquid nitrogen for microbial community analysis.

### 2.3. DNA Extraction, Library Construction, and Metagenomic Sequencing

Total genomic DNA was extracted from rumen fluid samples using the Mag-Bind^®^ DNA Kit (Omega Bio-tek, Norcross, GA, USA) following the manufacturer’s protocol. DNA concentration and purity were assessed with TBS-380 fluorometer (Turner BioSystems, Sunnyvale, CA, USA) and NanoDrop 2000 spectrophotometer (Thermo Fisher Scientific, Wilmington, DE, USA), respectively, and DNA quality was examined on a 1% agarose gel. DNA was fragmented to an average size of approximately 350 bp using a Covaris M220 (Gene Company Limited, Beijing, China) for paired-end library construction. Libraries were prepared using NEXTFLEX Rapid DNA-Seq (Bioo Scientific, Austin, TX, USA), with adapters containing full sequencing primer hybridization sites ligated to blunt-ended fragments. Paired-end sequencing was performed on an Illumina NovaSeq platform (Illumina Inc., San Diego, CA, USA) at Majorbio Bio-Pharm Technology Co., Ltd. (Shanghai, China) using the NovaSeq 6000 platform (Illumina, San Diego, CA, USA) with the NovaSeq 6000 S4 Reagent Kit v1.5 (300 cycles), following the manufacturer’s instructions.

### 2.4. Sequence Quality Control and Genome Assembly

Data analysis was performed on the Majorbio Cloud Platform (www.majorbio.com, accessed on 10 May 2026). Paired-end Illumina reads were trimmed to remove adapters and low-quality sequences (length < 50 bp or quality score < 20) using fastp (version 0.23.0) [16]. Metagenomic assembly was conducted using MEGAHIT (version 1.1.2) [17]. Contigs ≥300 bp were retained as final assembly results and used for subsequent gene prediction and annotation.

### 2.5. Gene Prediction, Taxonomy, and Functional Annotation

Open reading frames (ORFs) were predicted from assembled contigs using Prodigal 2.6.3 and MetaGene v1.0 [14,15]. ORFs ≥100 bp were retrieved and translated into amino acid sequences using the NCBI translation table. A non-redundant gene catalog was constructed using CD-HIT (version 4.6.1) [18] with 90% sequence identity and 90% coverage thresholds. High-quality reads were mapped to the gene catalog to calculate gene abundance using SOAPaligner (version 2.21) [19] with 95% identity.

### 2.6. Species and Functional Annotation

Representative sequences from the non-redundant gene catalog were aligned to the NR database using Diamond (version 0.8.35) with an e-value cutoff of 1 × 10^−5^ for taxonomic annotation. Cluster of Orthologous Groups (COG) annotations were assigned using Diamond against the eggNOG database with an e-value cutoff of 1 × 10^−5^. KEGG pathway annotation was performed using Diamond against the Kyoto Encyclopedia of Genes and Genomes database with the same e-value threshold. Carbohydrate-active enzymes were annotated using hmmscan against the CAZy database with an e-value cutoff of 1 × 10^−5^.

### 2.7. Statistical Analysis

After quality filtering, an average of 289,847,786,688 clean reads per sample were obtained for downstream metagenomic analyses. Low-quality reads and host-derived sequences were removed before assembly. Clean reads were assembled using MEGAHIT, v1.2.9, and open reading frames (ORFs) were predicted using MetaGeneMark v3.38. Predicted genes shorter than 100 bp were excluded from further analysis. A non-redundant gene catalog was constructed using CD-HIT v4.8.1 with a sequence identity threshold of 95% and coverage threshold of 90%. Functional annotation was performed against the KEGG, eggNOG, and CAZy databases using DIAMOND v2.0.15 with an e-value cutoff of 1 × 10^−5^. The best-hit alignment strategy was used for functional assignment. Statistical analyses were performed using SPSS 27.0 and R software (version 3.3.3). One-way ANOVA followed by Duncan’s multiple comparison test was used to analyze rumen fermentation parameters and alpha diversity indices after testing for normality and homogeneity of variance. Differences were considered significant at *p* < 0.05. Relative abundance data were normalized prior to downstream microbiome analyses to minimize the influence of sequencing depth differences among samples. Beta diversity was assessed based on Bray–Curtis distance matrices and visualized using non-metric multidimensional scaling (NMDS). Statistical significance among groups was evaluated using permutation multivariate analysis of variance (PERMANOVA) with 999 permutations. Redundancy analysis (RDA) was conducted to evaluate the relationships between rumen microbial composition and fermentation parameters. The significance of environmental variables was assessed using Monte Carlo permutation tests with 999 permutations. Data visualization was performed using R v4.3.1 (ggplot2) and GraphPad Prism v9.0.

## 3. Results

### 3.1. Effects of Different Concentrate-to-Roughage Ratios in Diets on Beta Diversity of Rumen Microorganisms

**Figure 1:**
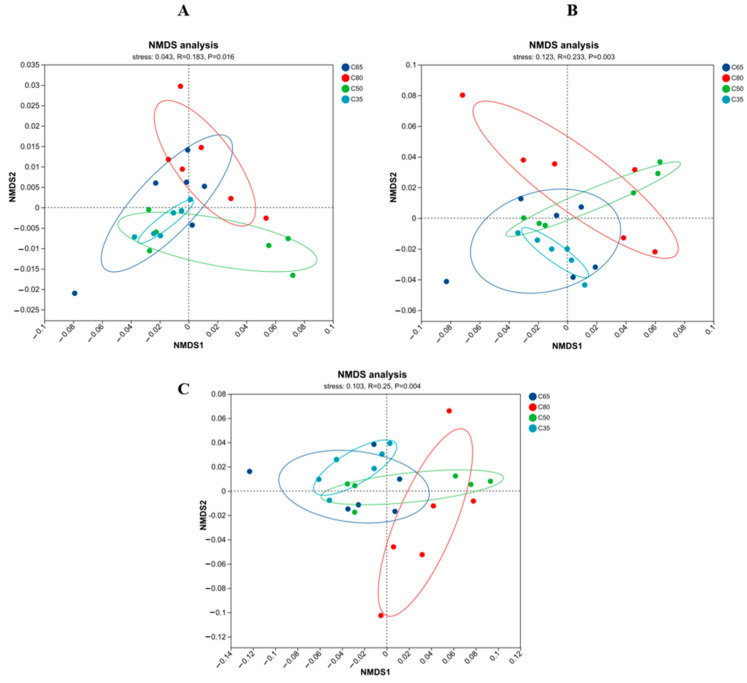
Microbial Beta diversity analysis based on Bray–Curtis distance matrix (NMDS analysis): (**A**) KEGG; (**B**) COG; (**C**) CAZy.

Non-metric multidimensional scaling (NMDS) based on the Bray–Curtis distance matrix was employed to evaluate differences in microbial functional structure among the four diets with varying concentrate-to-roughage ratios. As shown in Figure 1A, the microbial communities displayed clear separation across dietary groups at the KEGG pathway level (stress = 0.043, R = 0.183, *p* = 0.016). The high-concentrate group (C80) and the low-concentrate group (C35) formed distinct clusters, indicating substantial divergence in microbial functional composition in response to differences in dietary concentrate levels. At the COG functional category level (Figure 1B), intergroup separation was even more evident (stress = 0.123, R = 0.233, *p* = 0.003). Samples from the C80 group showed marked differentiation from the lower-concentrate groups. At the CAZy family level (Figure 1C), significant functional differences were also observed among the dietary treatments (stress = 0.103, R = 0.25, *p* = 0.004). In particular, samples from the C35 group clustered tightly, demonstrating a consistent distribution pattern, whereas those from the C80 group exhibited a distinct spatial arrangement in the ordination. NMDS analysis revealed a clear separation among dietary groups, indicating that dietary concentrate-to-roughage ratio strongly influenced rumen microbial community structure. High-concentrate diets appeared to promote starch-utilizing and propionate-producing bacteria, whereas low-concentrate diets favored fiber-degrading microbial populations.

**Figure 2:**
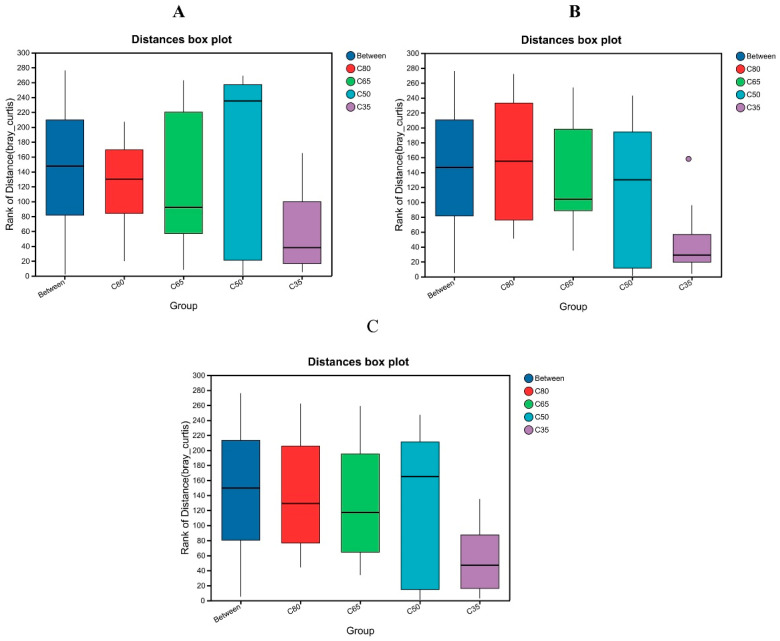
Microbial Beta diversity analysis based on Bray–Curtis distance matrix (ANOSIM analysis): (**A**) KEGG; (**B**) COG; (**C**) CAZy. Circles denote outliers (data points beyond 1.5 times the interquartile range from the box).

**Figure 3:**
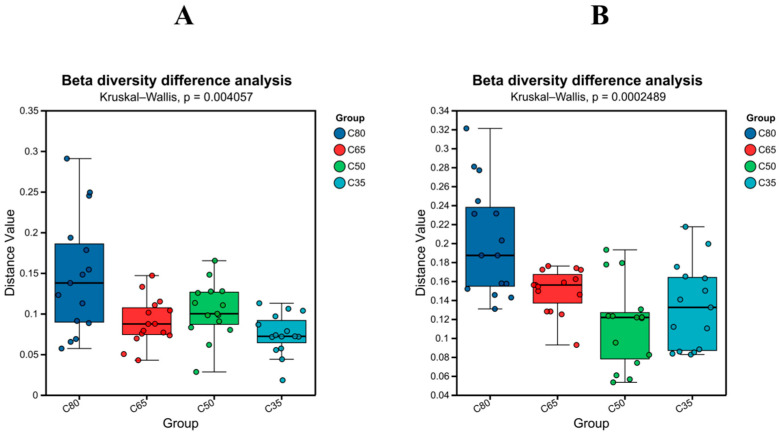
Based in the Bray–Curtis algorithm, an analysis was conducted to examine the inter-group differences in Beta diversity at different taxonomic levels of bacterial phyla. (**A**) Phylum; (**B**) genus.

To further verify differences in microbial functional structure among the dietary treatments, ANOSIM analysis based on the Bray–Curtis distance matrix was conducted. At the KEGG functional level (Figure 2A), intergroup distances exceeded intragroup distances, indicating significant differentiation in microbial metabolic profiles among the four diets. Notably, the C80 and C35 groups demonstrated clear functional separation, reflecting substantial variation in microbial metabolic characteristics across different concentrate-to-roughage ratios. At the COG functional category level (Figure 2B), a similar trend was observed, with intergroup variation consistently greater than intragroup variation. Functional categories associated with protein synthesis, carbohydrate metabolism, and energy-related processes exhibited greater variation in the C80 group, whereas the C35 group showed a more uniform distribution. At the CAZy family level (Figure 2C), the C35 group exhibited the largest intergroup differences and the lowest intragroup variability, indicating pronounced distinctions in carbohydrate-active enzyme profiles. To further quantify microbial community variation, β-diversity values among the four groups were compared using the Kruskal–Wallis test. At the phylum level (Figure 3A), β-diversity differed significantly among dietary treatments (*p* = 0.0041). The C80 group presented the highest degree of divergence, while the C35 group showed the lowest diversity variation. At the genus level (Figure 3B), differences among groups were even more pronounced (*p* = 0.00025), demonstrating that the increasing concentrate-to-roughage ratio was associated with greater variation in bacterial community composition.

### 3.2. Analysis of Microbial Species and Functional Composition in the Rumen of Cattle Fed Diets with Different Concentrate-to-Roughage Ratios

**Figure 4:**
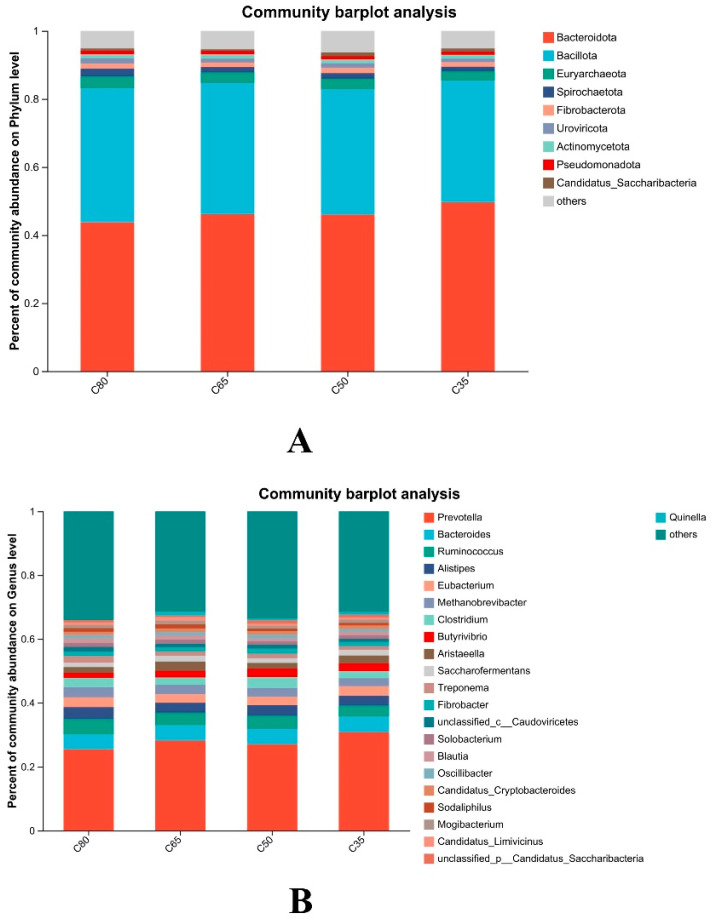
Analysis of species and functional composition of communities using the RPKM algorithm in the form of a column chart. (**A**) Phylum; (**B**) genus.

Phylum- and genus-level community composition analyses revealed marked differences in microbial community structure among the four dietary treatment groups. At the phylum level (Figure 4A), Bacteroidetes and Firmicutes (Bacillota) were predominant across all treatments, together accounting for more than 80% of total bacterial abundance. With increasing concentrate-to-roughage ratios, the relative abundance of Bacteroidetes showed an upward trend, whereas *Fibrobacterota* and Spirochaetota decreased. Under high-concentration feeding conditions, a slight reduction in Euryarchaeota (methanogenic archaea) was observed. At the genus level (Figure 4B), *Prevotella* was the dominant taxon, particularly in the C80 and C65 groups. In contrast, *Ruminococcus* and *Fibrobacter* were more abundant in the C35 group. The relative abundance of *Methanobrevibacter* showed a decreasing trend with increasing concentrate levels.

**Figure 5:**
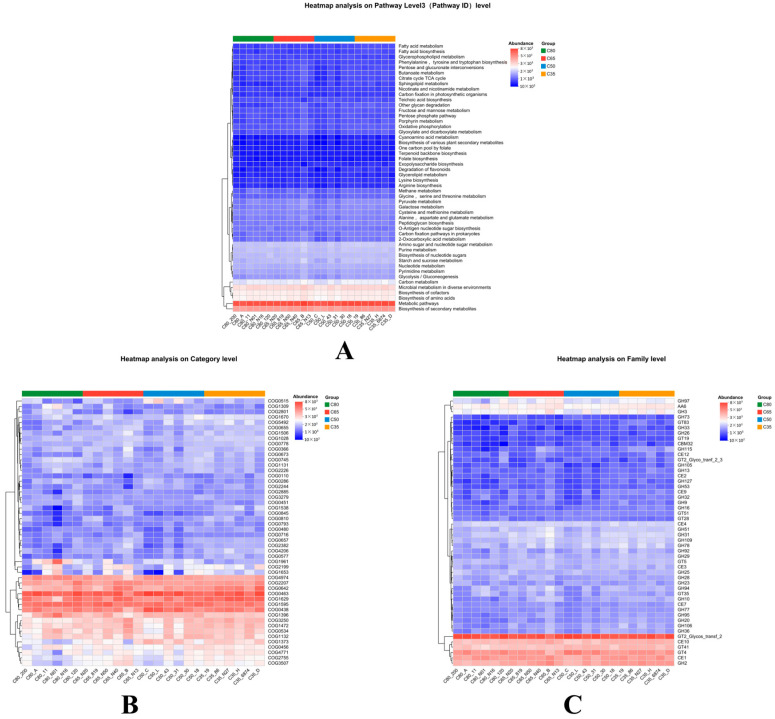
Heatmap analysis of KEGG Level 3 metabolic pathways (**A**), COG functional annotations (**B**), and carbohydrate-active enzyme (CAZyme) families (**C**) in the rumen microbiota under different dietary concentrate-to-roughage ratios.

Heatmap analysis further showed differences in microbial functional potential profiles across treatments (Figure 5). At the KEGG Level 3 pathway level (Figure 5A), high-concentrate groups (C80 and C65) were clearly separated from the lower-concentrate groups (C50 and C35). Pathways with higher relative abundance in the high-concentrate groups included glycolysis/gluconeogenesis, the pentose phosphate pathway, amino sugar and nucleotide sugar metabolism, starch and sucrose metabolism, pyruvate metabolism, propanoate metabolism, butanoate metabolism, fatty acid metabolism, and glycerophospholipid metabolism. Dietary interventions based on alternative feed resources have been shown to modulate rumen fermentation and reduce methane emissions through shifts in microbial activity [20]. In contrast, pathways located in the upper section of the heatmap—such as methane metabolism, carbon fixation in prokaryotes, nitrogen metabolism, oxidative phosphorylation, glyoxylate and dicarboxylate metabolism, porphyrin metabolism, and biosynthesis of secondary metabolites—exhibited relatively lower abundance or remained stable across treatments. At the COG functional classification level (Figure 5B), COG0519, COG1309, COG2801, COG1670, COG5492, and COG6505 showed higher relative abundance in the high-concentrate groups, whereas COG1538, COG0463, COG0480, COG0716, and COG1961 were more abundant under low-concentrate feeding conditions. At the carbohydrate-active enzyme (CAZy) family level, the high-concentrate groups exhibited increased abundance of the glycosyltransferase (GT) and auxiliary activity (AA) families, whereas glycoside hydrolase (GH) and carbohydrate esterase (CE) were more abundant in the low-concentrate groups. It should be noted that these results represent differences in functional gene abundance inferred from metagenomic data, rather than direct measurements of metabolic activity.

### 3.3. Analysis of Differences in Rumen Microbial Species and Function Between Diets with Different Concentrate-to-Roughage Ratios

**Figure 6:**
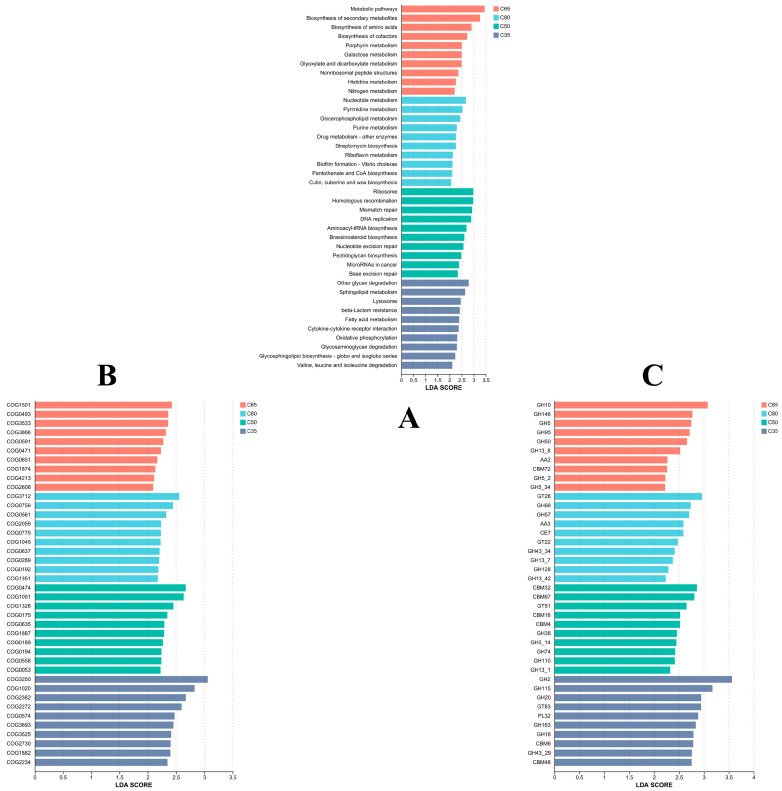
LefSe differential analysis of KEGG third-level metabolic pathways (**A**), COG functional annotations (**B**), and CAZyme families (**C**) in the rumen microbial community under different ratios of concentrate to roughage.

LefSe analysis further identified distinct functional biomarkers that differentiated the microbial communities among the four dietary treatments. At the KEGG Level 3 metabolic pathway level (Figure 6A), a series of pathways met the LDA > 2.0 threshold, demonstrating their strong discriminatory power across diets. The C80 group exhibited higher relative abundance in nucleotide metabolism, pyrimidine metabolism, and glycerophospholipid metabolism, all of which appeared prominently in the differential analysis. The C65 group showed enrichment in several major metabolic categories, including overall metabolic pathways, biosynthesis of secondary metabolites, biosynthesis of amino acids, biosynthesis of cofactors, and porphyrin metabolism. In contrast, the C50 group was characterized by enrichment in pathways such as ribosome, homologous recombination, mismatch repair, DNA replication, aminoacyl-tRNA biosynthesis, and peptidoglycan biosynthesis, which collectively distinguished this intermediate concentrate group from the others. The C35 group showed significant enrichment in pathways including fatty acid metabolism, oxidative phosphorylation, cytokine–cytokine receptor interaction, glycosaminoglycan degradation, and valine, leucine, and isoleucine degradation.

**Figure 7:**
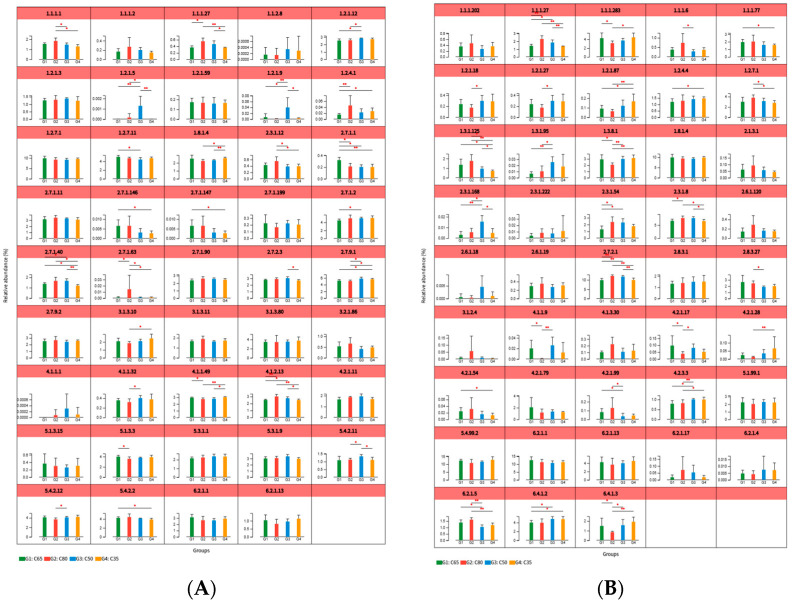
The inter-group differences in the glycolysis/glyoxylate (ko00010) metabolism (**A**) and propionic acid (ko00640) metabolism (**B**) pathways under different ratios of concentrated feed to roughage; * *p* < 0.05, ** *p* < 0.01.

At the COG functional classification level (Figure 6B), the C80 group was enriched in COG3712, COG0756, COG0561, and several additional functional categories that remained characteristic of the high-concentrate feeding condition. The C65 group exhibited higher abundance of COG1501, COG0493, COG3533, and others that collectively separated it from groups with lower concentration proportions. The C50 group showed enrichment in COG0474, COG1051, and COG1328, indicating distinctive functional features of this intermediate-diet microbial community. The C35 group was enriched in COG3520, COG1020, COG2382, and related categories. At the CAZy enzyme family level (Figure 6C), the C80 group showed enrichment in GT28, GH88, GH57, and several other CAZy families, which served as distinguishing functional markers for this treatment in the LEfSe output. Differences in enzyme abundance within the glycolysis/gluconeogenesis (ko00010) pathway (Figure 7A) showed that lactate dehydrogenase (EC 1.1.1.27), hexokinase (EC 2.7.1.1), and pyruvate kinase (EC 2.7.1.40) were significantly higher in the high-concentrate groups compared with the low-concentrate groups (C50 and C35) (*p* < 0.05). Conversely, enzymes associated with gluconeogenesis, such as phosphoenolpyruvate carboxylase (EC 4.1.1.49), were more abundant in the low-concentrate groups. In the propionate metabolism pathway (ko00640) (Figure 7B), propionyl-CoA carboxylase (EC 6.4.1.3) and succinyl-CoA synthetase (EC 6.2.1.5) exhibited significantly higher abundance in the high-concentrate groups, particularly in C80 (*p* < 0.05). In addition, enzymes such as acetyl-CoA synthase (EC 6.2.1.1) and methylmalonate semialdehyde dehydrogenase (EC 1.2.1.27) displayed relatively higher abundance in the C35 group.

### 3.4. Correlation Analysis of Rumen Microbial Fermentation Parameters and Environmental Factors in Diets with Different Concentrate-to-Roughage Ratios

According to the results presented in Table 2, the rumen pH of yaks in the C80 group was significantly below that of the C65, C50, and C35 groups (*p* < 0.05). In contrast, comparisons among the C65, C50, and C35 groups revealed no statistically significant differences (*p* > 0.05), indicating that these three dietary treatments produced relatively similar ruminal acidity conditions. Regarding ammonia nitrogen (NH_3_-N), the C35 group exhibited a significantly higher NH_3_-N concentration than the C65 group (*p* < 0.05). For rumen volatile fatty acids (VFAs), the effects of different concentrate-to-roughage ratios were not statistically significant across all treatment groups (*p* > 0.05), suggesting that total VFA concentration remained relatively stable despite changes in dietary composition.

**Figure 8:**
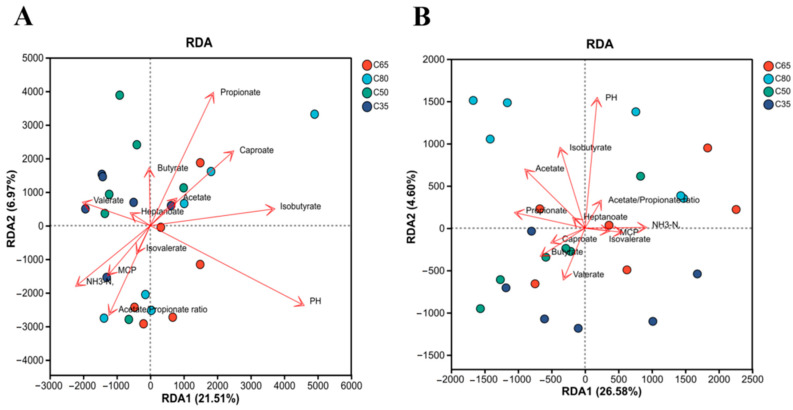
RDA of the relationship between dietary crude and fine components and rumen microorganisms and fermentation parameters in yaks. (**A**) Phylum level; (**B**) genus level.

To further elucidate how rumen microbial community structure corresponded with fermentation parameters under varying dietary concentrate-to-roughage ratios, a redundancy analysis (RDA) at the phylum level was conducted (Figure 8A). The first two principal axes, RDA1 and RDA2, jointly accounted for 28.48% of the variation observed in microbial community composition. As shown in the ordination diagram, samples from the four dietary treatments displayed a dispersed yet distinctly patterned distribution. Most samples from the C65 group were located on the right side of the RDA plot and exhibited strong positive correlations with propionate, caproate, isobutyrate, and pH. Conversely, samples from the C35 and C50 groups were primarily located in the left and upper-left regions of the diagram, clustering near variables such as butyrate, valerate, NH_3_-N, and microbial protein (MCP). Samples from the C80 group exhibited a more scattered distribution pattern; certain samples aligned near propionate and hexanoate, whereas others were positioned near variables such as NH_3_-N and the acetate-to-propionate ratio. At the genus level, redundancy analysis (Figure 8B) yielded results consistent with the phylum-level trends. The primary explanatory axis, RDA1, accounted for 26.58% of the total variation in community composition. In this analysis, samples from the C65 group were largely concentrated on the right side of the ordination plot and exhibited positive correlations with NH_3_-N and isovalerate. Samples from the C80 group were positioned predominantly above the coordinate axes and aligned closely with vectors representing pH and NH_3_-N. Meanwhile, samples from the C35 and C50 groups clustered on the left and lower-left regions of the plot and were associated with fermentation characteristics that included propionic acid, acetic acid, butyric acid, and valeric acid.

**Figure 9:**
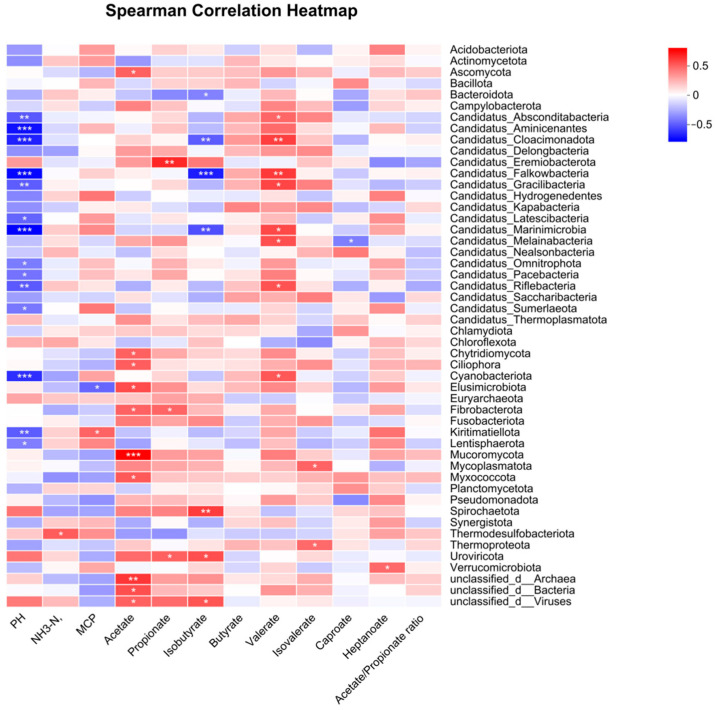
Heatmap analysis of the correlation between rumen microbial composition and fermentation parameters under different concentrate-to-roughage ratios in yak diets: phylum level.Significance levels: * *p* < 0.05, ** *p* < 0.01, *** *p* < 0.001.

To further clarify the relationships between dominant rumen fermentation parameters and bacterial community composition, a Spearman correlation heatmap was generated at the phylum level (Figure 9). A broad range of significant correlations of varying magnitudes was observed among the major phyla and key fermentation indicators. Bacteroidetes, recognized as the primary phylum involved in the degradation of cellulose and polysaccharides, exhibited a positive correlation with acetate. *Fibrobacterota*, another important fiber-degrading phylum, also demonstrated significant positive correlations with acetate and total VFA concentrations (*p* < 0.05). Euryarchaeota, consisting mainly of methanogenic archaea, showed significant positive correlations with acetate and pH (*p* < 0.05). Spirochaetota displayed significant positive correlations with acetate and highly significant positive correlations with isobutyrate (*p* < 0.01). These correlation patterns collectively highlight a structured set of associations between dominant microbial phyla and major fermentation parameters, illustrating measurable relationships across the dietary treatments without introducing interpretive conclusions beyond the presented data.

## 4. Discussion

The rumen microbial system exhibits high dynamism and responds sensitively to dietary composition, particularly the ratio of concentrate to roughage. It should be noted that the 24 h fasting procedure prior to slaughter may have partially influenced rumen fermentation characteristics and microbial composition; however, all animals were subjected to the same standardized sampling protocol to ensure comparability among treatments. β-diversity analysis indicates that rumen microbial community structure is significantly influenced by the concentrate-to-roughage ratio, a finding confirmed by both NMDS and ANOSIN analyses (Figure 1 and Figure 2). The high-concentrate-diet groups (C80, C65) and low-concentrate-diet groups (C50, C35) formed distinct communities, reflecting significant ecological differentiation driven by nutrient availability. High-concentrate diets provide abundant fermentable carbohydrates, lower rumen pH, and promote proliferation of amylolytic bacteria and propionate-producing bacteria. These microorganisms thrive in acidic, high-energy environments and enhance propionic acid and lactic acid production [2]. Conversely, low-concentrate diets characterized by higher fiber content and slower fermentation rates favored the growth of cellulolytic and acetate-producing bacteria [21]. Significant differences in β-diversity among dietary treatments revealed that increasing concentrate proportion not only altered microbial taxonomic composition but also compressed overall diversity ranges. At high energy levels, ecological redundancy may decrease, and excessive concentrate intake reduces microbial evenness while increasing fermentation-dominant microbial abundance—consistently with findings by Ramos et al. [22,23]. Under roughage-dominant diets, rumen microbial communities primarily degrade fiber and produce methane; high-concentrate diets favor starch fermentation and propionic acid production. This shift enhances energy conversion efficiency but may also increase risks of ruminal acidosis or dysbiosis if buffering capacity is insufficient [24,25]. Therefore, an appropriate amount of concentrate feed (e.g., C65) can balance efficient energy utilization with microbial stability, thereby maintaining a functionally diverse ruminal ecosystem.

Metagenomic analysis revealed significant changes in rumen microbial community composition at the phylum and genus levels in response to variations in the concentrate-to-roughage ratio of the diet. At the phylum level, the rumen microbiota was primarily composed of Bacteroidetes, Firmicutes, and Proteobacteria, consistently with previous reports in ruminants [3,26]. While methane mitigation strategies are increasingly explored, potential impacts on rumen metabolism and animal performance should also be carefully evaluated [27]. This relationship suggests that Bacteroidetes contribute to starch degradation and short-chain fatty acid production, whereas Firmicutes participate in cellulose and hemicellulose breakdown [8]. At the genus level, microbial communities were primarily composed of *Prevotella*, Bacteroides, and *Ruminococcus*. High-concentrate diets significantly increased the abundance of *Prevotella* and Bacteroides, which play key roles in soluble carbohydrate degradation, amino acid metabolism, and succinate–propionate fermentation pathways. Conversely, a high-roughage diet promoted the growth of *Ruminococcus*, *Fibrobacter*, and Clostridium butyricum, genera known for their cellulolytic capabilities and contributions to acetate and butyrate production. This shift toward a *Prevotella*-dominated community composition under high-concentrate feeding conditions signifies rumen adaptation to high-energy diets, promoting efficient starch utilization but potentially reducing fiber degradation efficiency [28].

Metagenomic functional annotation revealed distinct metabolic adaptations of rumen microbial communities to different concentrate levels in diets. Increasing the concentrate proportion significantly enhanced pathways associated with carbohydrate and energy production, while low-concentrate diets enriched functions related to fiber degradation, methane metabolism, and structural maintenance. At the KEGG Level 3 pathway level, high-concentrate diets (C80, C65) promoted enrichment in glycolysis/gluconeogenesis, starch and sucrose metabolism, and propionate metabolism, indicating enhanced carbohydrate fermentation and energy conversion. The increased propionate and pyruvate metabolism further indicates that fermentable starch increased the production of pyruvate-derived volatile fatty acids (VFAs), particularly propionate, which is a major precursor for host gluconeogenesis [29]. Conversely, grass-dominant diets (C50, C35) enriched pathways including methane metabolism, fatty acid degradation, nitrogen metabolism, and secondary metabolite biosynthesis—hallmarks of fiber-degrading, methanogenic communities adapted to structural carbohydrate substrates [30]. GOG functional classification confirmed these findings. High-concentrate diets stimulated gene categories associated with energy production and conversion, carbohydrate and amino acid transport and metabolism, reflecting enhanced catabolic activity and improved energy efficiency; low-concentrate diets exhibited microbial adaptation to complex fiber substrates and stable rumen environments. This pattern aligns with macro-genomic studies of ruminants by Silva [31] and Prabha [32]. CAZy family analysis further confirmed microbial enzyme system reorganization. The high-concentrate group promoted expression of glycosyltransferases and auxiliary activity enzymes responsible for starch and soluble sugar processing, whereas high-roughage feeding enriched glycoside hydrolases and carbohydrate esterases involved in cellulose and hemicellulose degradation [13]. This enzymatic profile pattern confirms that microbial communities modulate their carbohydrate-active enzyme composition based on substrate availability, shifting enzymatic activity from fiber degradation toward rapid carbohydrate fermentation with increasing concentrate levels [33]. The increased abundance of *Prevotella* in high-concentrate diets may be associated with its ability to degrade starch and non-fiber polysaccharides, suggesting enhanced adaptation to rapidly fermentable carbohydrate availability and increased propionate production potential. The higher abundance of *Ruminococcus* and *Fibrobacter* in low-concentrate diets indicates enhanced fiber-degrading capacity, which may contribute to improved cellulose and hemicellulose utilization and maintenance of rumen fermentation stability.

LefSE analysis further elucidated these metabolic differences. Enhanced amino acid, glyoxylate, and propionate metabolism in the high-concentrate group indicated increased microbial biosynthetic and energy-generating capacities. Conversely, the low-concentrate group exhibited enrichment in fatty acid metabolism and oxidative phosphorylation. These functional disparities reflect a metabolic trade-off between fermentation efficiency and ecological stability [3]. However, while high-concentrate diets enhance energy production, they may reduce microbial diversity and resilience, thereby increasing the risk of ruminal acidosis [34]. Overall, increasing concentrate ratios enhances microbial energy metabolism efficiency but may diminish ecosystem resilience, underscoring the importance of maintaining moderate concentrate levels to balance efficiency and stability. Analysis of enzyme profiles in glycolysis/gluconeogenesis (ko00010) and propanoic acid metabolism (ko00640) pathways revealed that elevated dietary concentrate ratios significantly alter rumen microbial energy metabolism. High-concentrate diets promoted increased relative abundance of key enzymes involved in carbohydrate degradation, glycolytic flux, and propionate synthesis, whereas low-concentrate diets favored expression of enzymes associated with gluconeogenesis and acetate production. Within glycolysis/gluconeogenesis pathways, elevated abundances of lactate dehydrogenase (EC 1.1.1.27), hexokinase (EC 2.7.1.1), and pyruvate kinase (EC 2.7.1.40) in the high-concentrate group indicated heightened glycolytic activity and rapid conversion of glucose to pyruvate, suggesting that carbohydrate-rich diets stimulate microbial degradation of starch-derived sugars, leading to increased production of pyruvate and its downstream fermentation products (e.g., propionic acid and butyric acid) [35]. In contrast, enzymes associated with gluconeogenesis, such as phosphoenolpyruvate carboxylase (EC 4.1.1.49), were more abundant in the low-concentrate group, indicating glucose synthesis under fiber-based feeding conditions [36]. Within the propionic acid metabolic pathway, the high-concentrate diet significantly enriched propionyl-CoA carboxylase (EC 6.4.1.3) and succinyl-CoA synthetase (EC 6.2.1.5), which are crucial for converting the propionic acid intermediate into succinyl-CoA—a key metabolite entering the tricarboxylic acid (TCA) cycle. This enrichment indicates that high-concentrate feeding enhances propionic acid production pathways via the succinate–propionate axis, thereby improving host energy acquisition efficiency. Conversely, the low-concentrate group exhibited higher activity of acetyl-CoA synthase (EC 6.2.1.1) and methylmalonate semialdehyde dehydrogenase (EC 1.2.1.27), enzymes associated with acetate and butyrate formation, suggesting a metabolic shift toward fiber fermentation with reduced energy yield [37,38]. These changes in enzyme activities align with predictions from KEGG and COG analyses.

It should be noted that all animals were subjected to a 24 h fasting period prior to rumen sampling. This procedure may influence rumen microbial composition and fermentation characteristics, including volatile fatty acid (VFA) concentrations. However, fasting was applied uniformly across all treatment groups to minimize variation caused by recent feed intake and to standardize sampling conditions at slaughter. Therefore, although fasting may have altered absolute fermentation values, the relative differences among dietary treatments remain comparable. Nevertheless, this factor should be considered when interpreting rumen fermentation profiles, and future studies under non-fasting or time-series sampling conditions are warranted to further validate these findings. The rumen is a digestive organ unique to ruminants, and a stable rumen environment is particularly crucial for these animals. pH, NH_3_-N, and VFAs are key indicators for maintaining rumen stability and reflecting fermentation status [39]. Typically, the optimal pH range for microbial growth and reproduction in the rumen is between 6.2 and 7.0. When rumen pH falls below 6.0, it may lead to subacute ruminal acidosis (SARA), inhibit fiber-degrading bacteria (such as *Ruminococcus* and *Fibrobacter*), thereby reducing crude fiber digestibility and inducing ruminal acidosis [40]. In this study, pH values in the C65, C50, and C35 groups ranged from 6.36 to 6.52, while the C80 group exhibited pH below 6.0, indicating a shift toward an acidic fermentation environment under high-concentrate feeding conditions. This pH decline was closely associated with the enrichment of *Prevotella*, *Succiniclasticum*, and Bacteroides. These genera showed significant positive correlations with propionic acid and VFA concentrations but negative correlations with pH [19]. These genera are typical starch-degrading and glycolytic bacteria that metabolize starch and soluble sugars via the succinate–propionate pathway. They consume hydrogen while producing large amounts of propionic acid and lactic acid, thereby reducing methane yield and improving energy recovery efficiency [41,42]. Conversely, the C35 group maintained higher pH levels, with *Ruminococcus*, *Fibrobacter*, and Butyrivibrio showing positive correlations with acetate. These fiber-degrading bacteria break down cellulose and hemicellulose, producing acetate and butyrate to form a slower yet more stable fermentation system [43]. The relationship between *Methanobrevibacter* and acetate in these groups further indicates that a certain association exists between cellulose-degrading bacteria and methanogens, achieved through interspecies hydrogen transfer. This process is crucial for maintaining methane production balance in fiber-fed yak [44]. NH_3_-N concentrations exhibited significant differences among dietary groups (*p* = 0.036), with the highest level in the C35 group (8.01 µg/mL) and the lowest in the C65 group (5.50 µg/mL). This suggests that excessive concentrate intake may reduce ammonia assimilation efficiency by altering microbial nitrogen metabolism [45]. These fermentation outcomes align with the enrichment of *Prevotella* and *Succiniclasticum* in high-concentrate groups and *Ruminococcus* and *Fibrobacter* in low-concentrate groups, which promote ammonia production through fiber degradation and microbial protein synthesis [46]. Redundancy analysis (RDA) further supported these relationships. Analysis of microbial composition at phylum and genus levels alongside key rumen fermentation parameters revealed that C80 and C65 communities tended toward regions of higher VFA concentrations and lower pH, confirming that the concentrate-to-roughage ratio in diets is a primary environmental factor influencing microbial structure and fermentation efficiency. Increasing concentrate ratios shifts the rumen toward a highly fermentative, low-pH system, favoring amylolytic bacteria and propionic acid metabolism; conversely, low-concentrate diets maintain a neutral, fiber-degrading, methane-producing system [47]. Collectively, these findings indicate that rumen microbial composition, functional potential, and fermentation environment are closely interrelated. High-concentrate diets promote carbohydrate fermentation and propionic acid production, enhancing energy conversion efficiency but carrying risks of acidosis instability. Conversely, low-concentrate diets preserve rumen buffering capacity and cellulose degradation capabilities, albeit at the expense of reduced energy yield.

Although the observed shifts in microbial composition, such as the enrichment of starch-degrading bacteria under high-concentrate diets and cellulolytic bacteria under high-roughage diets, are generally consistent with findings in other ruminant species, this study provides additional insights specific to yak, a unique ruminant adapted to the Qinghai–Tibet Plateau. The rumen microbiota of yak may exhibit distinct adaptive features due to long-term evolution under high-altitude, low-oxygen, and forage-limited environments. Furthermore, the present study integrates metagenomic functional potential with rumen fermentation characteristics, providing a more comprehensive understanding of how dietary concentrate-to-roughage ratio influences both microbial community structure and metabolic pathways. This functional-microbial integration at the gene-pathway level adds depth beyond taxonomic description alone and contributes to a systems-level understanding of rumen adaptation in yak. It should be noted that although this study focused on the dietary concentrate-to-roughage ratio, the experimental diets also differed in other nutritional components, including fiber fractions, fat content, mineral composition, and metabolizable energy. Therefore, the observed changes in rumen microbial composition, functional potential, and fermentation characteristics cannot be attributed exclusively to the concentrate-to-roughage ratio alone. These effects likely reflect the combined influence of multiple dietary factors. Future studies with more strictly controlled nutrient-matched diets are needed to further isolate the specific role of the concentrate-to-roughage ratio.

## 5. Conclusions

This study demonstrates that the dietary concentrate-to-roughage ratio is a key factor regulating the microbial composition, metabolic potential, and fermentation characteristics of the yak rumen. Increasing the concentrate proportion significantly altered microbial diversity and community composition, shifting the rumen ecosystem from a fiber-degrading and acetate-dominant type toward a carbohydrate-fermenting and propionate-dominant type. High-concentrate diets enriched starch-degrading and propionate-producing bacteria and enhanced pathways related to glycolysis (ko00010) and propionate metabolism (ko00640), while resulting in decreased rumen pH and altered fermentation patterns. In contrast, low-concentrate diets favored cellulolytic bacterial populations and maintained higher rumen pH and acetate production. Integration of metagenomic and fermentation data suggests that a moderate concentrate-to-roughage ratio (approximately 65:35) may provide a more balanced rumen microbial structure and fermentation profile under the conditions of this study. However, further validation incorporating growth performance, feed efficiency, and long-term physiological responses is required to determine the optimal dietary strategy. These findings provide mechanistic insights into how dietary energy density influences rumen microbial ecology and function, offering theoretical support for improving yak feeding strategies in plateau production systems.

## Figures and Tables

**Table 1:** Composition and nutrient level of experimental feed (dry matter basis).

Items	Groups			
	C80	C65	C50	C35
Ingredients				
Oat hay (%)	20	35	50	65
Corn (%)	36.5	27.5	16.7	6.5
Wheat (%)	9	6.8	5.7	4.3
Wheat bran (%)	8.5	8	7.3	7
Soybean meal (%)	7.6	5.5	5	3.5
Rapeseed meal (%)	9	8.2	7.3	6.7
Cottonseed meal (%)	6.4	6	5	4
Premix ^1^ (%)	2	2	2	2
NaCl (%)	1	1	1	1
Total	100	100	100	100
Nutrient level				
Metabolizable energy (ME/(MJ/kg) ^2^)	9.9	9.35	8.4	8.1
Dry matter (%)	84.1	83.0	80.3	75.8
Crude protein (%)	13.081	13.02	13.0	13.03
Crude ash (%)	4.94	5.58	6.33	7.04
Neutral detergent fiber (%)	61.5	60.3	54.1	59.2
Acid detergent fiber (%)	37.06	37.8	34.3	29.9
Crude fat (g/kg)	15.0	22.0	25.0	14.0
Ca (mg/kg)	286.0	507.0	524.0	396.0
P (mg/kg)	115.79	261.25	248.36	168.58

Note: ^1^ Each kg of premix contained Co at 0.20 mg, Cu at 15 mg, I at 0.80 mg, Fe at 100 mg, Mn at 25 mg, Se at 0.17 mg, Zn at 50 mg, VA at 3000 IU, VD at 350 IU, and VE at 40 IU. ^2^ The metabolic energy of nutrient level was calculated, and the rest was measured. The nutrient composition of the oat hay: CP: 10%; CF: 31%; ADF: 39%; NDF: 63%; EE: 2.3%; ASH: 8%; Ca: 0.40%; P: 0.27%. Nutrient levels reflect the total mixed diets (concentrate + oat hay). A separate analysis of the concentrate mixture was not performed in this study.

**Table 2:** Effects of dietary concentrate to coarse ratio on rumen fermentation in yaks.

Items	Groups				*p*-Value
	C80	C65	C50	C35	
pH	5.91 ± 0.18 ^a^	6.36 ± 0.12 ^b^	6.44 ± 0.13 ^b^	6.52 ± 0.12 ^b^	0.001
AA mmol/L	0.31 ± 0.03	0.25 ± 0.05	0.31 ± 0.06	0.27 ± 0.05	0.215
PA mmol/L	0.30 ± 0.07	0.29 ± 0.05	0.26 ± 0.07	0.24 ± 0.07	0.496
IBA mmol/L	0.28 ± 0.06	0.27 ± 0.07	0.25 ± 0.06	0.26 ± 0.05	0.894
BA mmol/L	0.28 ± 0.06	0.26 ± 0.04	0.30 ± 0.07	0.25 ± 0.05	0.555
VA mmol/L	0.28 ± 0.06	0.28 ± 0.05	0.30 ± 0.07	0.25 ± 0.06	0.994
IVA mmol/L	0.27 ± 0.06	0.26 ± 0.05	0.30 ± 0.06	0.26 ± 0.05	0.496
HA mmol/L	0.27 ± 0.08	0.26 ± 0.05	0.29 ± 0.08	0.25 ± 0.07	0.881
HPA mmol/L	0.25 ± 0.06	0.24 ± 0.05	0.25 ± 0.06	0.29 ± 0.05	0.488
VFA mmol/L	2.24 ± 0.50	2.11 ± 0.41	2.26 ± 0.53	2.07 ± 0.45	0.635
NH3-N(µg /mL)	7.05 ± 1.24 ^ab^	5.50 ± 0.95 ^b^	6.92 ± 1.25 ^ab^	8.01 ± 1.31 ^a^	0.036
Cpr (mg/mL)	8.98 ± 0.89	13.64 ± 2.71	11.02 ± 1.34	11.00 ± 4.20	0.385

Values with different superscript letters are significantly different.

## Data Availability

The original contributions presented in the study are publicly available. This data can be found here: Mendeley Data, Version 1, https://doi.org/10.17632/gzzch2nh7w.1.
